# Physician Quality Reporting System Program Updates and the Impact on Emergency Medicine Practice

**DOI:** 10.5811/westjem.2015.12.29017

**Published:** 2016-03-02

**Authors:** Jennifer L. Wiler, Michael Granovsky, Stephen V. Cantrill, Richard Newell, Arjun K. Venkatesh, Jeremiah D. Schuur

**Affiliations:** * University of Colorado, Department of Emergency Medicine, Aurora, Colorado; † George Washington University, Department of Emergency Medicine, Washington D.C.; ‡ CEP America, Department of Emergency Medicine, Emeryville, California; § Yale University School of Medicine, Department of Emergency Medicine and Center for Outcomes Research and Evaluation, New Haven, Connecticut; ¶ Brigham and Women’s Hospital, Harvard University School of Medicine, Department of Emergency Medicine, Boston, Massachusetts

## Abstract

In 2007, the Centers for Medicaid and Medicare Services (CMS) created a novel payment program to create incentives for physician’s to focus on quality of care measures and report quality performance for the first time. Initially termed “The Physician Voluntary Reporting Program,” various Congressional actions, including the Tax Relief and Health Care Act of 2006 (TRHCA) and Medicare Improvements for Patients and Providers Act of 2008 (MIPPA) further strengthened and ensconced this program, eventually leading to the quality program termed today as the Physician Quality Reporting System (PQRS). As a result of passage of the Affordable Care Act of 2010, the PQRS program has expanded to include both the “traditional PQRS” reporting program and the newer “Value Modifier” program (VM). For the first time, these programs were designed to include pay-for-performance incentives for all physicians providing care to Medicare beneficiaries and to measure the cost of care. The recent passage of the Medicare Access and Children’s Health Insurance Program (CHIP) Reauthorization Act in March of 2015 includes changes to these payment programs that will have an even more profound impact on emergency care providers. We describe the implications of these important federal policy changes for emergency physicians.

## INTRODUCTION

In 2001 the Institute of Medicine published a landmark review which noted that there were significant gaps in the quality of healthcare being delivered in the United States.^1^ Research over the past two decades has also shown substantial variations in, and relationship between, the cost of care delivered to Medicare beneficiaries and quality outcomes.^2^ These patterns emerged amidst a growing national concern that federal healthcare spending was increasing at an unsustainable pace, which threatens national bankruptcy.^3^ As such, the Centers for Medicaid and Medicare Services (CMS), the nation’s largest insurer, chose to launch a novel payment program named the Physician Voluntary Reporting Program (PVRP) designed to incent physicians to focus on quality of care measures. The goal of the PVRP program was to financially reward providers for successfully reporting a set of quality measures to CMS. The program required that a physician report their performance to CMS via administrative claims, or billing data, on a limited number of quality measures. From the initial implementation of the PVRP, emergency medicine was one of the medical specialties with the highest proportion of program participants.^4^ Over the last decade the program has been modified and expanded many times, eventually evolving into the current permanent CMS provider quality payment program termed the Physician Quality Reporting System (PQRS).^5^,^6^ Today the expanded PQRS program includes both the “traditional PQRS” reporting program, in addition to the newer “Value Modifier” program (VM). Described by CMS as “a reporting program that uses a combination of incentive payments and payment adjustments to promote reporting of quality information by eligible professionals (EPs),” this program includes evaluations of EP quality and cost measure performance and tiers providers based on this performance.^7^ Eligible professions are defined by CMS as physicians: doctor of medicine, doctor of osteopathy, doctor of podiatric medicine, doctor of optometry, doctor of dental surgery, doctor of dental medicine, doctor of chiropractic; practitioners: physician assistant, nurse practitioner, clinical nurse specialist, certified registered nurse anesthetist, certified nurse midwife, clinical social worker, clinical psychologist, registered dietician, nutrition professional, audiologist or therapist, physical therapist, occupational therapist, and qualified speech-language therapist. Importantly, the definition of EPs includes all part-timers, moonlighters, and other episodic providers who were registered in Medicare’s Provider Enrollment, Chain, and Ownership System (PECOSs) as of October 15, 2015.^8^ The Accountable Care Act requires that CMS officially transition the VM program to a penalty program in 2015.

### PQRS Updates

On October 31, 2014, the CMS released its 2015 Medicare Physician Fee Schedule Final Rule, which detailed changes to the federal quality reporting requirements for payment of physician services.^9^ Beginning in 2015 the penalties for non-participation in the PQRS programs (both the “traditional PQRS” and VM program) become more significant and compounded. Physician groups of 10 or more EPs that choose not to participate in any of the PQRS programs are subject to a maximum penalty of 6% of Medicare payments (Table 1). This represents a potential 2% withheld for not successfully reporting via the “traditional PQRS” program and an additional 4% automatic penalty under the VM program (2% for individual physicians and those in groups of less than 10 eligible providers). The definition of a “group” is defined by use of the same tax identification number (TIN) by EPs. CMS also announced that it intends to publicly report physician performance rates for all PQRS measures collected in 2016 (based on 2015 performance) on the “Physician Compare” website.

The American Board of Emergency Medicine (ABEM) promoted an additional 0.5% incentive for Medicare fee for service work by attesting to participation in the 2014 PQRS program. There is no ABEM incentive currently in place for performance year 2015 or beyond.

#### Traditional PQRS Program (2005–2015)

To avoid the 2% penalty, EPs must participate in the traditional PQRS program and report performance on established quality measures. To date, there are currently five ways to report performance for participation in the PQRS programs for emergency physicians (Table 2a and 2b). These include direct submission (i) via an electronic health record (EHR) product of certified health information technology vendor, (ii) via the CMS Group Practice Reporting Option (GPRO) web interface, (iii) a CMS “qualified” registry, (iv) a qualified clinical data registry (QCDR), or (v) claims (billing) data. The first two options do not tend to be viable for most small single specialty independent emergency physician practices. Submission by an EHR is not often practical because as a hospital-based specialty, the EHR vendor administration is typically not managed by the emergency medicine physician group but rather the hospital. The GRPO reporting process is a viable option for hospital-employed or larger multispecialty groups, which according to CMS was used by roughly 5,500 EM providers in 2014.^10^ New in 2015 is the requirement that groups choosing to report via GPRO must administer and report patient experience survey data (Consumer Assessment of Healthcare Providers and Systems, CAPHS) at the groups’ expense. Option (iii), qualified registries are those that have been reviewed and approved by CMS. Very few exist specific to only EM.^11^ The option to report via a QCDR is currently limited to a single large group practice on the west coast, which has the only fully functional private EM-specific QCDR today. This option, however, will be more available after the American College of Emergency Physicians implements their version.^12^ Until then, as of today most emergency physicians report via claims data.

One aspect of the PQRS program is the notable lag between the performance and payment periods. Specifically, dollars paid (or penalties) in 2015 for physician services are based on a two-year “look back.” Meaning that in 2015, payment for services to Medicare beneficiaries is based on how a provider on quality measures in 2013. This is also true for the VM Program. Therefore, the reported data are unlikely to be actionable for quality improvement nor allow patient consumers timely assessments to make care utilization decisions. The 0.5% bonus offered for participation in the PQRS Maintenance of Certification program (as that offered by ABEM) expired after 2014, and performance of these quality improvement activities in not set to be publically reported.

In 2015, CMS retired 50 quality measures including four of the five that were previously commonly reported by EM providers as part of the 2014 “emergency care cluster.” These include PQRS #28: Aspirin for AMI, #55: 12-Lead Electrocardiogram for syncope, #56: Pneumonia (community-acquired pneumonia): Vital Signs, and #59: Pneumonia (CAP): Empiric Antibiotic. The list of remaining measures potentially applicable to EM is limited (Table 3). Claim submissions are denoted by the addition of PQRS codes, which are abstracted by an EP’s coding and billing company and then placed in the claim submission form. To avoid the payment adjustment, in 2015 individuals must report nine measures across three National Quality Strategy (NQS) domains with at least one “cross-cutting” measure. This new list of cross-cutting measures represents a core set where CMS believes that there are significant performance gaps across specialties. The only measure that applies to typical emergency care, albeit not easily, is PQRS #317 “Preventive Care and Screening: Screening for High Blood Pressure and Follow Up Documented” (NQS Community-Population Health Domain). The measure specifications state that this is to apply to all Medicare patients who have a documented emergency department (ED) systolic blood pressure greater than 120 or diastolic greater than 80.^13^

Groups are required to report on nine measures across three domains. A performance score of zero does not satisfy the requirements. Given these requirements and the available measures pertinent to EM, it is unlikely that the typical individual emergency physician practice will be able to satisfy the reporting requirements. As such, most will be subject to the Measure Applicability Validation (MAV) process. Through this process CMS groups PQRS measures into measure clusters. CMS expects that if a provider reports on one measure in a cluster that the provider could report on additional measures within the same cluster. CMS reviews the provider’s claims to see if the provider could have reported on additional measures within the cluster. If CMS finds that the provider could have reported additional measures within the cluster but did not, the provider will be deemed as failing the MAV process and a PQRS payment adjustment may apply. If CMS does not find additional measures within the cluster that the provider could have reported on, the provider will be deemed as passing the MAV. CMS established an alternative option for satisfying reporting for emergency physicians by defining an “emergency medicine cluster” (Table 4). It is recommended that the typical EM provider should select this reporting option.

Qualified clinical data registries (QCDRs) are an alternative PQRS reporting option. QCDRs are certified registries of quality metrics that allow providers to report on a different set of measures than those in PQRS. The measures in QCDRs must be approved by CMS, but they do not require National Quality Forum (NQF) approval, streamlining the measure development process. Although not an option for EM in the past, in 2015 CMS approved two QCDRs for EM reporting.^14^ QCDRs may submit information on both PQRS and up to 30 non-PQRS specialty-specific measures. This methodology does not require reporting of a cross-cutting measure or measure endorsement by the National Quality Forum (NQF) process nor does it require participation in the CAHPS program. It does require data collection and submission for all payers and allows for a more comprehensive view of a specialists practice and collection of measures of rare events and diagnosis as the sample size is not limited to Medicare patients. In addition, first-year QCDR measures are not considered in the calculation of the VM quality component given the lack of historical benchmark data. The measures for the EM American College of Emergency Physicians (ACEP) QCDR have not been finalized, but a potential set of measures have been developed (Table 5). There is a plan to have potential measures released for public comment later this summer.

#### Value Modifier Program

Section 3007 of the Affordable Care Act (ACA) mandates that CMS begin applying a VM payment adjustment, based on cost and quality metrics, to physician payments starting in 2015.^15^ It also requires that the modifier be added in a budget-neutral manner. This means that within the national performance pool there must be winners and losers in the program. Ranking will be done with primary designated specialties, so emergency providers will compete against themselves. A similar but distinct VM program has been in existence for hospital (facility) performance since 2013. The VM program is based on performance in two main categories: quality and cost. Quality tiering is based on six defined quality of care domains (clinical care, patient experience, population/community health, patient safety, care coordination, and efficiency measures). Cost tiering is based on performance on five per capita cost measures; total per capita costs (Parts A and B) and total per capita costs for beneficiaries with four chronic diseases (diabetes, cornary artery disease, chronic obstructuve pulmonary disease, and heart failure). These cost measures are separated into two per capita domains: total overall costs measure and total costs for beneficiaries with specific conditions (four measures). Payment for the VM is based on overall quality and cost performance, as compared to a benchmark, and depends on the practice size an EP is associated with (Table 6a and 6b).

The benchmarks for 2015 VM performance are based on 2014 performance. A national mean is calculated by including all physician groups with 100 or more EPs. Quality measures that are new in the performance period are not benchmarked in the quality composite calculation during the following one year. Medicare Spending Per Beneficiary (MSPB) costs are the sum from three days before to 30 days after index admission. Attribution is given to those who charged the most Medicare Part B (provider) charges during the index inpatient stay. The EM codes (99281-99285) are exempt from attribution.

Participation in the VM program is similar to those for PQRS (GPRO, traditional registry, EHR or claims submissions). Physician VM payments for 2015 excluded physicians who provide services in rural health clinics, federally qualified health centers, critical access hospitals (CAHs), and groups physicians participate in Medicare Shared Savings Program Accountable Care Organizations (ACOs), pioneer ACOs, and Comprehensive Primary Care Initiatives. However, these groups are included in 2015 performance for 2017 VM payments. During this same performance period nurse practitioners, physician assistants and clinical nurse specialists’ costs will be attributed to their associated TIN. The VM program requires participation in the traditional PQRS program (described above). Failure to participate in the PQRS program will affect both traditional PQRS payments (-2%), in addition to VM payments (−4%) for a maximum of a −6% penalty for groups with 10 or more.

Patient attribution for EP performance is based on a retrospective assignment based on claims. The methodology is the same as Medicare Shared Savings Program assignment to an Accountable Care Organization.^16^ Patient assignment is to a group or individual TIN based on following cascading prioritization: (i) plurality of evaluation and management (E&M) primary care visits, then (ii) plurality of E&M specialty care if no primary care. Emergency medicine billing codes (CPT 99281-99285) are exempt from attribution methodology, but urgent care codes are not. This assignment to a provider is invisible to patients and there are no patient penalties for behaviors that drive costs.

## WHAT DOES THE FUTURE OF FEDERAL PROVIDER MEASUREMENT PROGRAMS HOLD FOR EMERGENCY MEDICINE?

In April 2015 the Medicare Access and Children’s Health Insurance Program (CHIP) Reauthorization Act (MACRA) was passed by Congress.^17^ This bill not only repealed the Sustainable Growth Rate (SGR) (which was used to calculate physician fee for service payments), it also directs that the current PQRS programs (i.e. VM and traditional PQRS) programs will continue through 2018. However, starting in 2019 a new program titled the Merit-Based Incentive Payment System (MIPS) will be initiated. This novel program increases at-risk Medicare provider payments to up to 9% (plus or minus) by 2022. Assessment categories dictated by law are in the stated categories of quality, resource use, EHR meaningful use, and clinical practice improvement activities. Those who participate in, and receive a significant share of their revenues through “alternative payment models,” will be exempt from the MIPS program.

Alternative payment programs (APM) have yet to be fully specified. Until the regulations are written, it is unclear exactly what the impact will be on EM. However, it is critical that EM begins to develop model programs that may be a way to generate innovative payment models which describe the value of high quality emergency care services. Recent work facilitated by the Brookings Institute that described the need for payment innovation for acute care services is an important first step in this development.^18^ QCDR measures should align with this APM model.

Hospitals have a growing number of required quality reporting programs that are similar to, but distinctly different from, the provider-based PQRS program. These include measures described within the Outpatient Quality Reporting, Inpatient Quality Reporting, Value-Based Payment, and Core Measure. CMS has been clear that it intends to increase the amount of money at risk for provider performance. Now MACRA defines that at least 20% of physician’s Medicare payments will be at risk in the next decade. Continuing to research to evaluate the opportunities for emergency medicine to show economic value to the system are critical for our specialty.

## Figures and Tables

**Table 1:** Summary of physician quality reporting system program impact on 2015 reporting and 2017 payments.*

	2014	2015
PQRS		
Bonus for traditional PQRS+	+0.5% payment in 2015	No incentives
Bonus for PQRS maintenance of certification+	+0.5% payment in 2015	
Penalty for failure to satisfy PQRS	−2.0% in 2016	−2.0% in 2017
Value modifier		
Additional penalty for failure to satisfy PQRS	−2.0% in 2016	Up to −4.0% in 2017
Total potential maximum penalties	−4.0% in 2016	−6.0% in 2017

* Increasing impact of physician quality reporting system (PQRS) participation.

**Table 2a:** 2015 Physician quality reporting system (PQRS) reporting options.*

Reporting mechanism	Measure type	Reporting criteria	Applicability to emergency medicine
Claims	Individual measures	Report at least 9 measures covering at least 3 National Quality Strategy (NQS) domains, including 1 cross-cutting measure, and report each measure for at least 50% of the Medicare Part B fee for service (FFS) patients seen during the reporting period to which the measure applies. If less than 9 measures apply, report 1–8 measures covering 1–3 NQS domains, but subject to Measures Applicability Validation Process (MAV). Measures with a 0 performance rate will not be counted.	Viable option. Only option for cross cutting measure applicable to emergency medicine (EM) is #317 – Screening for high blood pressure and follow up documented.
Qualified registry	Individual measures	Report at least 9 measures covering at least 3 NQS domains OR, if less than 9 measures covering at least 3 NQS domains apply, report 1–8 measures covering 1–3 NQS domains, AND report each measure for at least 50% of the Medicare Part B FFS patients seen during the reporting period to which the measure applies. If less than 9 measures apply, report 1–8 measures covering 1–3 NQS domains, but subject to MAV. Measures with a 0 performance rate will not be counted.	Viable option.
Qualified registry	Measures groups	Report at least 1 measures group, and report each measures group for at least 20 patients, the majority (11 patients) of which must be Medicare Part B FFS patients. Measures groups containing a measure with a 0 percent performance rate will not be counted.	Not viable option. Measure group specifications for minimum participation do not allow most individuals to successfully report based on low volumes.
Direct electronic health record (EHR) product or EHR data submission vendor	Individual measures	Report 9 measures covering at least 3 of the NQS domains. If an eligible professional’s (EP’s) EHR product/vendor does not contain patient data for at least 9 measures covering at least 3 domains, then the EP would be required to report all of the measures for which there is Medicare patient data. EPs are required to report on at least 1 measure for which there is Medicare patient data.	Typically not viable.
Qualified Clinical Data Registry (QCDR)	Individual PQRS and/or non-PQRS measures	Report at least 9 measures available for reporting under a QCDR covering at least 3 of the NQS domains, and report each measure for at least 50% of all applicable patients (both Medicare and non-Medicare). Of these measures, at least 2 must be outcome measures, or, if 2 outcomes measures are not available, at least 1 outcome measure and at least 1 resource use, patient experience of care, efficiency/appropriate use, or patient safety measure.	Will be viable in 2016.

* Option for individual physicians.

**Table 2b:** 2015 Physician quality reporting system (PQRS) reporting options.*

Group practice specifications	Measure type	Reporting mechanism	Reporting criteria
2–99 Eligible professionals (EPs)	Individual measures	Qualified registry	Report at least 9 measures covering at least 3 National Quality Strategy (NQS) domains, including 1 cross-cutting measure, and report each measure for at least 50% of the Medicare Part B fee for service (FFS) patients seen during the reporting period to which the measure applies. If less than 9 measures apply, report 1–8 measures covering 1–3 NQS domains, but subject to Measures Applicability Validation Process (MAV). Measures with a 0 performance rate will not be counted.
	Individual measures and CAHPS for PQRS	Direct EHR product or EHR data submission vendor product and use of Centers for Medicare & Medicaid Services (CMS) certified survey vendor	The group practice must have all Consumer Assessment of Healthcare Providers and Systems (CAHPS) for PQRS survey measures reported on its behalf via a CMS-certified survey vendor, and report at least 6 additional measures, outside of CAHPS for PQRS, covering at least 2 of the NQS domains using the direct EHR product or electronic health record (EHR) data submission vendor product. If less than 6 measures apply to the group practice, the group practice must report up to 5 measures. Of the additional 6 measures that must be reported in conjunction with reporting the CAHPS for PQRS survey measures, a group practice would be required to report on at least 1 measure for which there is Medicare patient data.
25–99 Eligible professionals	Individual group practice reporting option (GPRO) measures in GPRO web interface	GPRO web interface	Report on all measures included in the web interface; and populate data fields for the first 248 consecutively ranked and assigned beneficiaries in the order in which they appear in the group’s sample for each module or preventive care measure. If the pool of eligible assigned beneficiaries is less than 248, then group practice must report on 100% of assigned beneficiaries. Must report on at least 1 measure for which there is Medicare patient data.
25–99 EPs, OR ≥100 EPs	Individual GPRO measures in the GPRO web interface and CAHPS for PQRS	GPRO web interface and use of CMS certified survey vendor	Requires CAHPS be completed for PQRS survey measures reported on its behalf via a CMS-certified survey vendor. Report on all measures included in the GPRO Web Interface (as above).
	Individual measures and CAHPS for PQRS	Qualified registry and use of CMS certified survey vendor	The group practice must have all CAHPS for PQRS survey measures reported on its behalf via a CMS-certified survey vendor, and report at least 6 additional measures, outside of CAHPS for PQRS, covering at least 2 of the NQS domains using the qualified registry. If less than 6 measures apply to the group practice, the group practice must report up to 5 measures. Of the additional measures that must be reported in conjunction with reporting the CAHPS for PQRS survey measures, the group practice must report on at least 1 measure in the cross-cutting measure set.

* Group reporting options.

**Table 3:** Potential physician quality reporting system (PQRS) reporting measures for emergency care.

PQRS#	NQS domain	Quality measure title	Reporting mechanism	MAV cluster
#54	Clinical effectiveness	EM:12-lead ECG performed for non-traumatic chest pain	Claims Registry	Claims: cluster 4 Registry: none
#76	Patient safety	Prevention of CRBSI: central venous catheter insertion protocol	Claims Registry	Claims: cluster 12 Registry: cluster 24 *can report alone
#91	Clinical effectiveness	Acute otitis externa (AOE): topical therapy	Claims Registry	Claims: cluster 7 Registry: cluster 12
#93	Efficiency	AOE: systemic antimicrobial therapy – avoidance of inappropriate use	Claims Registry	Claims: cluster 7 Registry: cluster 12
#187	Clinical effectiveness	Stroke & stroke rehabilitation: thrombolytic therapy (tPA)*	Registry	Registry: cluster 21
#254	Clinical effectiveness	Ultrasound determination of pregnancy location for pregnant patients with abdominal pain	Claims Registry	Claims: cluster 4 Registry: none
#255	Clinical effectiveness	Rh immunoglobulin (Rhogam) for Rh-negative pregnant women at risk of fetal blood exposure	Claims Registry	Claims: cluster 4 Registry: none
#317	Community-population health	Preventative care and screening: screening for high blood pressure and follow up documented	Claims Registry	Claims: cross cutting Registry: cross cutting
#326	Clinical effectiveness	Atrial fibrillation and atrial flutter: chronic anticoagulation therapy†	Claims Registry	Claims: none Registry: none

*NQS,* national quality strategy; *MAV,* measures applicability validation process; *EM,* emergency medicine; *ECG,* electroencephalogram; *CRBSI,* catheter-related bloodstream infection

* Also known as hospital STK-4.

† Also known as STK-3.

**Table 4:** 2015 Emergency medicine cluster.

Title	PQRS #	Domain	Description
Cluster 4			
Emergency care	54	Effective clinical care	Emergency medicine: 12-lead electrocardiogram (ECG) performed for non-traumatic chest pain
	254	Effective clinical care	Ultrasound determination of pregnancy location for pregnant patients with abdominal pain
	255	Effective clinical care	Rh immunoglobulin (Rhogam) for Rh-negative pregnant women at risk of fetal blood exposure
Cross-cutting	317	Population & community health	Preventative care and screening: screening for high blood pressure and follow-up documented

Note: Cross-cutting measures represents a core set where Centers for Medicaid and Medicare Services (CMS) believes that there are significant performance gaps across specialties. Measure #317 is the only measure that applies to emergency care patients as defined by the measure specifications. Because most emergency physicians will be subject to the Measure Applicability Validation (MAV) because of a limited number of attributable quality measures, CMS created a Emergency Medicine cluster. If eligible professionals use this cluster they will pass the MAV process.

**Table 5:** Potential qualified clinical data registries (QCDR) physician quality reporting system (PQRS) quality measures.

PQRS#	Measure title	NQS domain
#54	12-lead electroencephalogram (ECG) performed for non-traumatic chest pain	Clinical effectiveness
#76	Prevention of catheter-related bloodstream infections (CRBSI): central venous catheter insertion protocol	Patient safety
#91	Acute otitis externa (AOE): topical therapy	Clinical effectiveness
#93	Acute otitis externa (AOE): systemic antimicrobial therapy–avoidance of inappropriate use	Clinical effectiveness
#187	Stroke and stroke rehabilitation: thrombolytic therapy (tPA); also known as hospital STK-4	Clinical effectiveness
#254	Ultrasound determination of pregnancy location for pregnant patients with abdominal pain	Clinical effectiveness
#1	ED utilization of CT for minor blunt head trauma for patients aged 18 years and older	Efficiency & cost reduction
#2	ED utilization of CT for minor blunt head trauma for patients aged 2 through 17 years	Efficiency & cost reduction
#3	Coagulation studies in patients presenting with chest pain with no coagulopathy or bleeding	Efficiency & cost reduction
#4	Appropriate ED utilization of CT for pulmonary embolism	Efficiency & cost reduction
#5	ED LOS for discharged ED patients–overall rate	Patient experience of care
#6	ED LOS for discharged ED patients–general rate=(overall rate – psych patients – transfer patients)	Patient experience of care
#7	ED LOS for discharged ED patients–psych mental health patients	Efficiency & cost reduction
#8	ED LOS for discharged ED patients–transfer patients	Efficiency & cost reduction
#9	Door to diagnostic evaluation by a qualified medical personnel	Patient safety
#10	Anti-coagulation for acute pulmonary embolism patients	Patient safety
#11	Pregnancy test for female abdominal pain patients	Patient safety
#12	Three-day return rate for ED visits	Communication & care coordination
#13	Three-day return rate for UC visits	Communication & care coordination
#14	Tobacco screening and cessation intervention for asthma and COPD patients	Effective clinical care
#15	tPA considered	Community-population health
#16	Adult sinusitis: antibiotic prescribed for acute sinusitis	Efficiency & cost reduction
#17	Adult sinusitis: appropriate choice of antibiotic	Efficiency & cost reduction
#18	Avoidance of antibiotic treatment in adults with acute bronchitis	Efficiency & cost reduction

*NQS,* National Quality Strategy; *ED,* emergency department; *CT,* computed tomography; *LOS*, length of stay; *UC,* urgent care

**Table 6a:** Calculation of the 2017 value modifier using the quality-tiering approach.†

Cost/quality	Low quality	Average quality	High quality
Low cost	0.0%	+2.0x*	+4.0x*
Average cost	−2.0%	0.0%	+2.0x*
High cost	−4.0%	−2.0%	0.0%

† Groups with >10 eligible professionals.

* Groups eligible for an additional +1.0% (if average beneficiary risk score in the top 25% of all beneficiary risk scores where “x” represents the upward payment adjustment factor. The upward payment adjustment factor will be determined after the performance period has ended based on the aggregate amount of downward payment adjustments).

**Table 6b:** Calculation of the 2017 value modifier using the quality-tiering approach.†

Cost/quality	Low quality	Average quality	High quality
Low cost	0.0%	+1.0x*	+2.0x*
Average cost	0.0%	0.0%	+1.0x*
High cost	0.0%	0.0%	0.0%

† Groups with 2–9 eligible professionals and solo practitioners.

* Groups eligible for an additional +1.0% (if average beneficiary risk score in the top 25% of all beneficiary risk scores where “x” represents the upward payment adjustment factor. The upward payment adjustment factor will be determined after the performance period has ended based on the aggregate amount of downward payment adjustments).
